# Research progress on specific and non-specific immune effects of BCG and the possibility of BCG protection against COVID-19

**DOI:** 10.3389/fimmu.2023.1118378

**Published:** 2023-01-31

**Authors:** Jingli Du, Yue Su, Ruilan Wang, Enjun Dong, Yan Cao, Wenjuan Zhao, Wenping Gong

**Affiliations:** Tuberculosis Prevention and Control Key Laboratory/Beijing Key Laboratory of New Techniques of Tuberculosis Diagnosis and Treatment, Senior Department of Tuberculosis, The 8th Medical Center of PLA General Hospital, Beijing, China

**Keywords:** Bacille Calmette-Guerin (BCG), COVID-19, specific and non-specific immunity, tuberculosis (TB), trained immunity

## Abstract

Bacille Calmette-Guérin (BCG) is the only approved vaccine for tuberculosis (TB) prevention worldwide. BCG has an excellent protective effect on miliary tuberculosis and tuberculous meningitis in children or infants. Interestingly, a growing number of studies have shown that BCG vaccination can induce nonspecific and specific immunity to fight against other respiratory disease pathogens, including SARS-CoV-2. The continuous emergence of variants of SARS-CoV-2 makes the protective efficiency of COVID-19-specific vaccines an unprecedented challenge. Therefore, it has been hypothesized that BCG-induced trained immunity might protect against COVID-19 infection. This study comprehensively described BCG-induced nonspecific and specific immunity and the mechanism of trained immunity. In addition, this study also reviewed the research on BCG revaccination to prevent TB, the impact of BCG on other non-tuberculous diseases, and the clinical trials of BCG to prevent COVID-19 infection. These data will provide new evidence to confirm the hypotheses mentioned above.

## 1 Introduction

Tuberculosis (TB) is still the most common cause of death for single hereditary pathogens. According to the Global Tuberculosis Report 2022 released by the World Health Organization (WHO), there were an estimated 10.6 million new TB cases and an estimated 1.6 million deaths in 2021 (1). China remains one of the 30 high TB burden countries, with an estimated 842,000 new TB cases in 2020, ranking second and accounting for about 9% of new cases globally (2).

Bacille Calmette-Guérin (BCG), developed by Calmette and Guerin by attenuating the virulence of *Mycobacterium bovis* in 1908, is a century-old vaccine for TB prevention (3). BCG has an excellent protective effect on miliary tuberculosis and tuberculous meningitis in children or infants, but its protective efficacy in adults varies between 0% - 80% (4, 5). In 1995, the WHO did not recommend BCG revaccination because of the lack of sufficient scientific evidence on the protective efficiency induced by BCG revaccination, and the Chinese Health Commission also stopped the work related to BCG revaccination in adults in 1997 (6). Interestingly, a growing number of studies have demonstrated that BCG could induce a nonspecific memory immunity termed “trained immunity” in innate immune cells by activating higher frequencies of innate immune cells to secrete interleukin 1β (IL-1β), tumor necrosis factor α (TNF-α), and IL-6 cytokines (5, 7–12). Therefore, the hypothesis has been proposed that BCG-induced trained immunity can protect against infection with microorganisms other than *Mycobacterium tuberculosis* (M. tb) (13, 14).

The coronavirus disease 2019 (COVID-19) pandemic, caused by severe acute respiratory syndrome coronavirus 2 (SARS-CoV-2), has been raging worldwide for nearly three years. As of 13 January 2023, there were 661,545,258 confirmed cases of COVID-19 and 6,700,519 deaths worldwide (https://covid19.who.int/). More than ten COVID-19-specific vaccines have been approved for emergency use worldwide, such as Sinovac, Sinopharm BBIBP-CorV, Ad5-nCoV vaccine, ZF2001, Janssen’s Ad26.COV2.S, Pfizer-BioNTech’s BNT162b2, Moderna mRNA-1273, Gam-COVID-Vac (Sputnik V), EpiVacCorona vaccine, AstraZeneca’s ChAdOx1 nCoV-19 (AZD1222), CureVac, and BBV152 vaccines (10, 12, 15–26). However, with the emergence of new SARS-CoV-2 variants of concern (VOC), the protective efficiency of these vaccines is facing unprecedented challenges (16). Previous studies have suggested that BCG might be a potential candidate to compensate for the shortcomings of COVID-19-specific vaccines (4, 10–12, 27–29).

In this study, we summarized specific and nonspecific immune responses induced by the BCG vaccine, the effect of BCG revaccination on the incidence of TB and other diseases, and the effects of BCG vaccination against COVID-19 infection.

## 2 Immune responses induced by the BCG

BCG is the only approved vaccine for preventing TB, and the target population is infants and newborns. BCG can reduce the risk of developing the disease by 50% and has a better preventive effect on severe tuberculosis (30). In addition, it can effectively prevent tuberculous meningitis and disseminated tuberculosis in infants. BCG is a live vaccine made from Mycobacterium bovine tuberculosis bacilli. After vaccination, it can activate T lymphocytes to produce specific immune responses (31). Since 1921, BCG has been used to prevent TB in humans, and since 1974, the WHO has included it in the Expanded Program on Immunization (EPI) (31). In recent years, research has found that the BCG vaccine can stimulate our body to produce atopic immunity, mediate innate immunity, and provide non-specific immunity. Innate immunity is the initial barrier against infection by pathogenic microorganisms, which can produce nonspecific immune responses to different pathogens, such as chemotaxis, phagocytosis, and secreted cytokines. It has long been believed that innate immunity differs from adaptive immunity and does not have immune memory. However, more and more studies have shown that innate immune cells and their stem cells also have memory characteristics (32), indicating that the innate immune responses have been activated after receiving the first infection. Then it can produce a more robust immune responses after being infected again to provide a better protective effect in fighting against pathogens (32). After being activated by homologous or heterologous pathogens, the innate immune system can produce a more robust immune response, and this phenomenon is called “trained immunity” (33). It is worth noting that not all heterologous/homologous exposures lead to innate immune training, on the contrary, some exposures can induce the immunoparalysis of innate immune cells (34).

### 2.1 Specific immune responses induced by the BCG

Although BCG remains the world’s only vaccine against TB, the duration of its preventive effect on TB is limited. Its efficacy in children with tuberculous meningitis and miliary tuberculosis is consistent, but its effectiveness in adults with pulmonary TB is variable (35). Possible reasons are as follows: 1) Genetic variation in the BCG strains, such as Denmark/Copenhagen strain 1331, Russian/Moscow, and Japanese/Tokyo 172; 2) The immune response to the BCG vaccine is weakened in the elderly due to immune aging; 3) Non-tuberculosis mycobacteria (NTM) infection masked the true effect of the BCG vaccine against M.tb; 4) Continuous stimulation of BCG can only induce more short-lived effector memory T cells and effector T cells (36–38).

**Figure 1:**
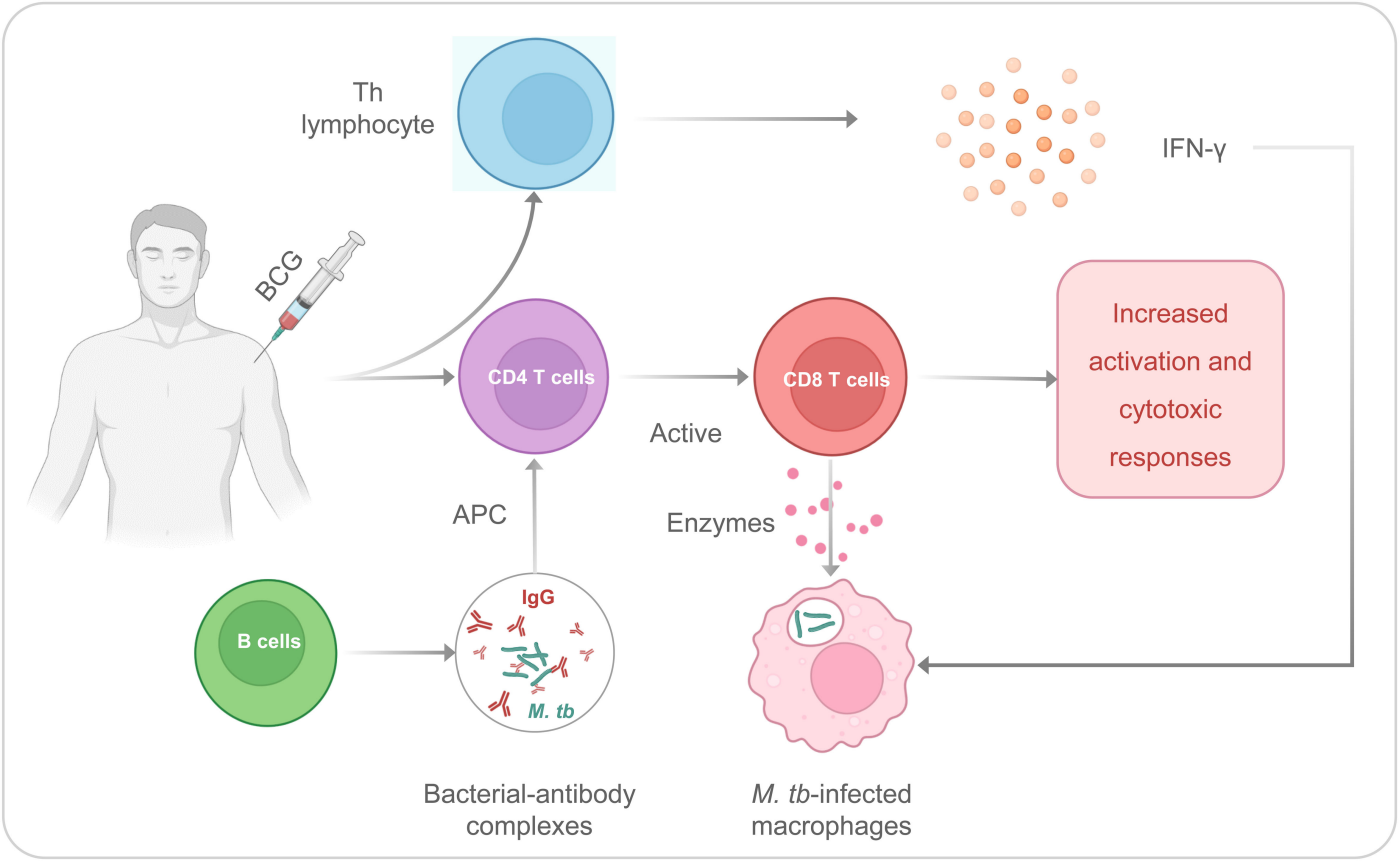
The Th and CD4/CD8 T cells are contributed to the adaptive immune responses induced by the BCG.

It has been observed that BCG could induce specific or adaptive immunity to fight against infection. The T helper (Th) lymphocyte response and CD4/CD8 T cells are contributed to the adaptive immune response induced by the BCG (39, 40) (**Figure 1**). The Th cells can secrete interferon γ (IFN-γ), and the BCG immunization after birth induces the production of IFN-γ by γδ T cells. It is necessary for protective immunity from tuberculosis because this cytokine increases antimycobacterial activity in macrophages (41). In addition, CD4 and CD8 T cells would be considered primary mediators for long-term immunity for M. tb infection. CD8 T cells could identify mycobacterium-infected macrophages, and these macrophages are attacked by enzymes secreted by CD8 T cells, which directly protect against M. tb (40). After immunization with BCG, B cell activation in serum increases, and IgG against M. tb increases significantly (42, 43). These antibodies can also form bacterial-antibody complexes that can induce increased M. tb processing and antigen presentation by antigen-presenting cells (APCs) to CD4T cells, leading to increased CD8 T cell activation and cytotoxic responses to M. tb (43, 44).

### 2.2 Nonspecific immune responses induced by the BCG

**Figure 2:**
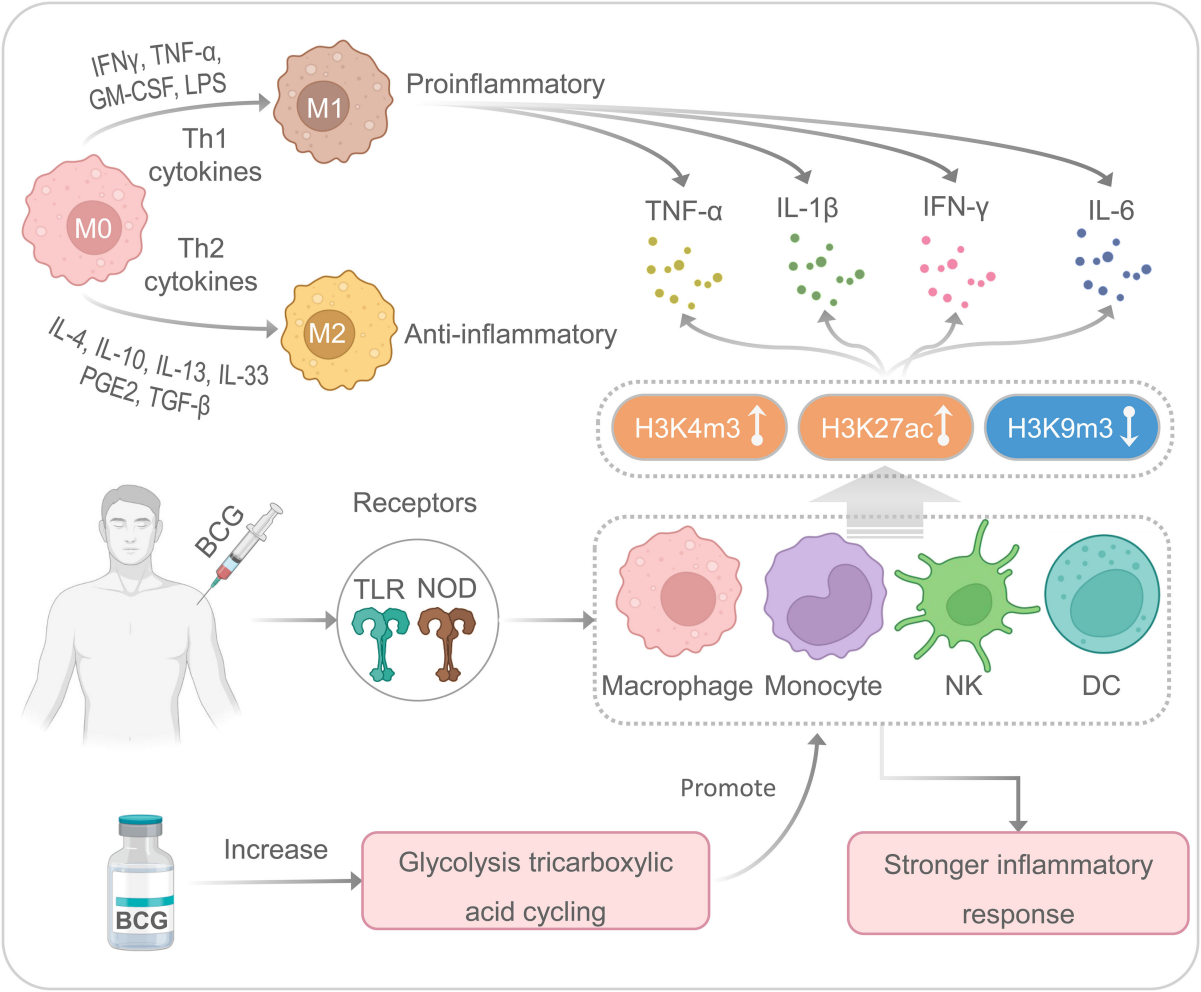
The nonspecific immune responses induced by the BCG. M, macrophages; NK, natural killer cells; DC, dendritic cells.

BCG is a multifunctional vaccine with nonspecific or heterologous immunomodulatory effects, which can promote nonspecific immunomodulatory effects (45) (**Figure 2**). Our previous studies have indicated that mature myeloid cells, such as macrophages or monocytes, play an essential role in nonspecific immunity induced by the BCG vaccine against unrelated pathogens (4, 12, 27). Although it has previously been shown that BCG-induced immunity is compartmentalized, trained innate immunity induced at a bone-marrow level in their respective hematopoietic stem cells, recent studies have indicated that BCG vaccination can also induce trained innate immunity in tissue-resident macrophages such as alveolar macrophages and peritoneal macrophages (46). BCG primary immunization induces more open chromatin structure in macrophages or monocytes, increases transcriptional active histone modifications such as H3K4m3 (histone H3 trimethylation at lysine 4) and H3K27ac (histone H3 acetylation at lysine 27), and decreases transcriptional inhibitory histone modifications such as H3K9m3 (histone H3 trimethylation at lysine 9), which in turn activates the production of IL-1β, TNF-α, and IL-6 (9, 12, 33, 47). Interestingly, when infected with an unrelated pathogen such as SARS-CoV-2, these trained nonspecific immune cells can induce a quicker and more substantial increase of H3K27ac and H3K4m3 and a decrease of H3K9m3, resulting in a higher level of IL-1β, TNFα, and IL-6 (33, 48). It also has been reported that BCG-mediated nonspecific immunity involves toll-like receptors (TLR) on the cell membrane surface and nucleotide-binding oligomerization domain (NOD) receptors located in the cytoplasm of some nonspecific immune cells, such as macrophages, monocytes, natural killer (NK) cells, and dendritic cells (DCs) (49, 50). The activation of TLRs and NOD receptors initiates the inflammatory cascade mainly through the secretion of TNF-α, IL-1β, and IL-6 (48, 51, 52). These cytokines aggregate inflammatory cells and provide signals to innate immune cells (53).

Moreover, enhanced glycolysis and glutamine-driven tricarboxylic acid cycling have been proven to be an essential metabolic pathway for trained immunity induced by BCG (54). There are two main types of macrophage polarization: classical polarization (M1) and alternative polarization (M2) (55). M1 macrophages are usually involved in eliminating pathogens and limiting tumor growth, while M2 macrophages are mainly involved in pathological processes such as anti-inflammatory response, tissue healing, fibrosis, and tumor survival (56). It has been suggested that lipopolysaccharide (LPS) and Th1 cytokines (IFN-γ, TNF-α, and GM-CSF) can stimulate M1-type polarization to produce pro-inflammatory cytokines (IL-1β, IL-6, IL-12, and IL-23), while Th2 cytokines, such as IL-4 and IL-13, can stimulate M2-type polarization to secrete anti-inflammatory cytokines (IL-10 and TGF-β) (57, 58). Interestingly, macrophages are highly plastic innate immune cells, and M1 and M2 macrophages can transform into each other based on different microenvironmental stimuli (59). In addition, M1 macrophages exert robust cytotoxic and antiproliferative effect activity by producing reactive oxygen species and nitric acid reactive substances (60, 61). However, recent studies have shown enhanced glycolysis and increased lactic acid production during M1 polarization (62). Several studies suggest that BCG can induce macrophages to secrete cytokines (such as IL-6, IL-12, and TNF-α) and a novel macrophage activated associated protein 1 (NMAAP1) (59, 63). Furthermore, BCG immunization can induce enhanced glucose consumption and lactate production *via* Akt/mTOR pathway (54).

## 3 Effect of BCG revaccination on the incidence of TB

Although the immune effects of BCG are not lifelong, BCG is still an important measure to control TB. The WHO recommends that BCG should be given as early as possible after the baby’s birth in countries with an endemic and high incidence of TB. It has been suggested that BCG revaccination could induce a more robust trained immunity defense against TB or other diseases (64) (**Table 1**). A previous study assessed the effectiveness and cost-effectiveness of BCG revaccination among adolescents, and the results showed that revaccination reduced the likelihood of TB transmission, with the risk of infection falling from 5.7% per year to 4.8% per year (65). In addition, in cost-benefit evaluations, if the cost of revaccination per person is set at $1-10, the effectiveness of the vaccine is between 10% and 80%, the term of protection is ten years, and the cost of treatment for the vaccinated population to restore healthy life (disability-adjusted life year, DALY) per year is $116 to $9237. The intervention was about twice as cost-effective as preventing transmission, with recovery costs of US $52 to US $4,540 per DALY at 80% effectiveness and 17% of cases averted with revaccination. According to the survey, BCG revaccination is cost-effective compared to international benchmarks (65).

**Table 1:** The research of revaccinating BCG to protect against tuberculosis.

Authors (Countries)	Study Design	Results
Christopher Dye South Africa	9290 new cases observed in a cohort	(1) Reduce the likelihood of TB transmission (2) Risk of infection falling from 5.7% to 4.8% per year (3) Cost-effectiveness: about twice as cost-effective as preventing transmission
Rakshit S, Ahmed A, Adiga V, Sundararaj BK, Sahoo PN, Kenneth J, et al. India	Two hundred healthy adults, BCG vaccinated at birth, were tested for their IFN-γ release assay (IGRA) status. Of these, 28 IGRA+ and 30 IGRA– were BCG revaccinated, and 24 IGRA+ and 23 IGRA– subjects served as unvaccinated controls.	(1) IFN-γ and/or IL-2 Ag85A- and BCG-specific CD4+ and CD8+ T cell responses were boosted by revaccination at 4 and 34 weeks (2) CD4+ T cells expressing up to 8 cytokines were also significantly enhanced in both IGRA+ and IGRA- vaccinees relative to unvaccinated controls, most markedly in IGRA+ vaccinees. (3) Compared with control group, the innate IFN-γ+ NK/γδ/NKT cell responses were higher in both IGRA+ and IGRA- vaccinees. (4) the immunogenicity of BCG was not affected even in patients with LTBI
Nemes E, Geldenhuys H, Rozot V, et al. South Africa	Randomized, three-arm, placebo-controlled, partially-blinded clinical trial aimed to enroll 990 healthy, HIV-uninfected, QFT-negative, 12- to 17-year-old adolescents, BCG- vaccinated in infancy	An effectiveness of 45.4% for continuous QFT conversion by BCG reinoculation

Furthermore, BCG revaccination may benefit individuals with latent tuberculosis infection (LTBI). An Indian study showed that after BCG revaccination, the response of Ag85A and BCG-specific CD4^+^ and CD8^+^ T cells were significantly enhanced. Furthermore, the re-inoculated BCG was immunogenic in both positive and negative subjects with interferon release tests, suggesting that the immunogenicity of BCG was not affected even in patients with LTBI (66). It was also shown that Ag85A-, BCG-and LTG-specific CD4^+^ and CD8^+^ T cell responses were more enhanced in individuals with positive interferon release tests than in individuals with negative interferon release tests (66). Therefore, BCG revaccination may benefit people with LTBI who are more likely to develop the disease. Another randomized controlled trial conducted in South Africa employed the QuantiFERON-TB Gold In-tube test to assess the protective effects of BCG revaccination, and the result showed an effectiveness of 45.4% for continuous QFT conversion by BCG reinoculation (67).

BCG revaccination is linked to a trained innate immune mechanism. For example, a clinical trial conducted in South African adults with latent tuberculosis infection (LTBI) showed that BCG revaccination promoted BCG-specific CD4^+^CD8^+^γδ T cells and enhanced the responses of BCG-reactive CD3^+^CD56^+^ NKT-like cells and CD3^-^CD56^dim^IFN-γ^+^ or CD^3-^CD56^hi^ IFN-γ^+^ NK cells (68). Similarly, a phase 1b randomized study performed in South African adolescents observed the same outcome that BCG revaccination elicited robust, polyfunctional BCG-specific CD4^+^ T cells (69).

Although these studies suggest that BCG revaccination can protect against TB and LTBI, other studies have yielded different results. A randomized controlled trial assessed the effect of BCG revaccination on all-cause mortality in 46,889 individuals in Malawi, and the results showed that, with a 30-year follow-up, BCG revaccination did not have any beneficial effects on all-cause mortality (70). Therefore, whether BCG revaccination can bring benefits needs to be further studied and verified.

## 4 Effect of BCG vaccine on other diseases

### 4.1 Effect of BCG on respiratory diseases

The trained immunity of BCG vaccination showed a more robust and longer-lasting effect (9). These functions effectively prevent TB and protect against other infectious diseases, especially some respiratory diseases (9) (**Table 2**). Studies have demonstrated that BCG plays a protective role in fighting against respiratory syncytial virus (RSV) infection. RSV is the most common cause of lower respiratory diseases. It is the leading cause of hospitalization, morbidity, and mortality from respiratory infections, usually causing bronchiolitis and pneumonia in infants under six months, as well as rhinitis and colds in adults (71). RSV infections typically occur in 3% – 7% of the elderly, with respiratory symptoms more severe than influenza in this population (72, 73). Unfortunately, there is no licensed effective vaccine for RSV worldwide. Animal experiments have shown that a recombinant BCG vaccine (rBCG-N-hRSV) has endowed mice with protective immunity and enhanced their ability to challenge infection (74). On day 28 after BCG immunization, it was found that the mice in the BCG immunized group lost less body weight, revealed less neutrophil infiltration, and showed a lower viral load in bronchoalveolar lavage fluid than those in the control group (74). In addition, activated banks of T cells that release IFN-γ and IL-1 were found in spleen T cells, suggesting that this recombinant BCG vaccine could induce a mixed CD8 and CD4 T cell response to inhibit the viral spread and prevent lung damage (74, 75). Therefore, this recombinant BCG vaccine (rBCG-N-hRSV) is a promising candidate for preventing RSV infection.

**Table 2:** Effect of BCG on other diseases rather than TB.

Disease	Authors	Research	Result
Respiratory Syncytial Virus (RSV)	Céspedes PF, Rey-Jurado E, Espinoza JA, et al.	A single, low dose of a cGMP recombinant BCG vaccine elicits protective T cell immunity against the human respiratory syncytial virus infection and prevents lung pathology in mice	(1) Mice showed less weight loss and less infiltration of neutrophils and less viral load in bronchoalveolar lavage compared with the unimmunized controls. (2) Activated banks of T cells that release IFN-γ and IL-1 were found in spleen T cells. (3) This recombinant BCG vaccine (rBCG-N-hRSV) is a promising vaccine candidate for the prevention of RSV infection.
H_1_N_1_	Leentjens J, Kox M, Stokman R, et al.	BCG Vaccination Enhances the Immunogenicity of Subsequent Influenza Vaccination in Healthy Volunteers: A Randomized, Placebo-Controlled Pilot Study	(1) The combination of BCG vaccine and influenza vaccine was effective in preventing influenza virus infection. (2) Compared with the placebo group, the BCG-vaccinated trial group had a significantly stronger response to H1 antibodies to the influenza A(H1N1) vaccine strain and had a more rapid seroconversion trend.
	Mukherjee S, Subramaniam R, Chen H, Smith A, Keshava S, Shams H	Boosting efferocytosis in alveolar space using BCG vaccine to protect host against influenza pneumonia	(1) BCG vaccine could significantly enhance the efferocytosis effect of alveolar phagocytes (AP). (2) The efferocytosis effect of AP in mice after receiving BCG immunization was significantly enhanced. (3) All mice that received BCG injections were able to survive the deadly influenza A virus.
Bladder Cancer	Petar B, J WA, N GG	Old instillations and new implications for bladder cancer: the urinary microbiome and intravesical BCG	BCG immunotherapy has been the “gold standard” for the treatment of non-muscle invasive bladder cancer.
Leprosy	Glynn JR, Fielding K, Mzembe T, Sichali L, Banda L, McLean E, et al.	BCG revaccination in Malawi: 30-year follow-up of a large, randomized, double-blind, placebo-controlled trial	BCG vaccines were about 40% protected against leprosy in 30 years.
HPV	(1) Salem A, Nofal A, Hosny D (2) Podder I, Bhattacharya S, Mishra V, Sarkar TK, Chandra S, Sil A, et al.	(1) Treatment of Common and Plane Warts in Children with Topical Viable Bacillus Calmette-Guerin (2) Immunotherapy in viral warts with intradermal Bacillus Calmette-Guerin vaccine versus intradermal tuberculin purified protein derivative: A double-blind, randomized controlled trial comparing effectiveness and safety in a tertiary care center in Eastern India	(1) 65% of children with common warts were effectively treated with BCG as compared to the placebo group. (2) BCG vaccinations were given at 4 weeks intervals, and after treatment, the complete clearance of viral warts was 48.5%.
Type 1 Diabetes	(1) Keefe RC, Takahashi H, Tran L, Nelson K, Ng N, Kühtreiber WM, et al. (2) Kühtreiber WM, Faustman DL	(1) BCG therapy is associated with long-term, durable induction of Treg signature genes by epigenetic modulation (2) BCG Therapy for Type 1 Diabetes: Restoration of Balanced Immunity and Metabolism	Patients with type 1 diabetes who received at least 2 BCG vaccinations returned to normal blood glucose levels after about 3 years, and therapeutic effects were observed even in patients with advanced diabetes who had been ill for more than 20 years, and the therapeutic effect lasted for more than 5 years.
Atherosclerosis	van Dam AD, Bekkering S, Crasborn M, van Beek L, van den Berg SM, Vrieling F, et al.	BCG lowers plasma cholesterol levels and delays atherosclerotic lesion progression in mice	BCG reduced plasma total cholesterol levels in mice in the experimental group, reduced foam cell formation in peritoneal macrophages, delayed the progression of atherosclerotic lesions at the root of the aortic, and reduced the severity of lesions.

The influenza A virus epidemic that occurred in 2009 brought a huge disease burden on global health. A randomized, placebo-controlled pilot study conducted in 2015 showed that the combination of BCG and influenza vaccine effectively prevented influenza virus infection (76). In brief, the individuals vaccinated with the BCG vaccine had a significantly stronger response to H1 antibodies against the influenza A (H_1_N_1_) vaccine strain and had a more rapid seroconversion trend than those in the placebo group (76). Furthermore, the pro-inflammatory leukocyte response is enhanced after BCG vaccination, and a non-specific effect of influenza vaccines has also been observed, which can modulate cytokine responses to unrelated pathogens, suggesting that BCG vaccination before influenza vaccination may improve influenza vaccination efficiency (76, 77). Another animal experiment showed that the BCG vaccine could significantly enhance the efferocytosis effect of alveolar phagocytes (AP) (77). AP is crucial in maintaining lung health, and its funerary effect prevents pathogenic acute lung injury caused by lung infections. The efferocytosis effect of AP was significantly enhanced in mice immunized with the BCG vaccine, which increased the uptake and digestion of AP by apoptotic cells and initiated the rapid clearance of alveolar apoptotic cells (77).

Recently, a clinical trial was conducted on elderly people to evaluate the protective efficacy of BCG against respiratory infections (78). The primary endpoints of the study were time to first infection and incidence of new infections (respiratory infections caused by viruses, community-acquired pneumonia, hospital-acquired pneumonia, intra-abdominal infections, urinary tract infections, bloodstream infections, and acute bacterial infections of skin and skin structures). The results showed that the time to first infection in the BCG group was significantly delayed compared to the placebo group (16 weeks after BCG vaccination and 11 weeks in the placebo group). The incidence of new infections also showed a noticeable reduction, with 42.3% in the placebo group and 25.0% in the BCG group. The risk of new infections was reduced by 45% in the BCG group compared to the placebo group. Especially in preventing respiratory infections caused by viruses, participants in the BCG group had a 79% lower risk of viral pneumonia infection (78).

### 4.2 Effect of BCG on other infectious diseases

In addition to respiratory infectious diseases, BCG may also have a specific protective effect on other contagious diseases. For example, it was reported that BCG has a positive therapeutic effect on skin and genital warts caused by the human papillomavirus (HPV). A comparative study performed in Egypt showed that 65% of children with common warts were effectively treated with BCG compared to the placebo group (79). In a similar clinical trial conducted in India, three doses of BCG vaccinations were given at four weeks intervals, and after treatment, the complete clearance of viral warts was 48.5% (80). At the same time, some animal experiments have confirmed that the BCG vaccine can also resist various DNA and RNA virus infections (81). One study showed that children who received BCG immunization at birth had lower mortality rates than children who delayed BCG vaccination, which can be attributed to the BCG vaccine’s prevention of neonatal sepsis, respiratory infections, and fever (82, 83).

### 4.3 Effect of BCG on noninfectious diseases

Besides infectious diseases, BCG has also shown a protective effect against noninfectious diseases, such as non-muscle-invasive bladder cancer (NMIBC), type 1 diabetes mellitus (T1DM), and atherosclerosis (**Table 2**). Since the 1970s, BCG immunotherapy has been the “gold standard” for treating NMIBC (84). A randomized, double-blind, randomized controlled clinical trial showed that the protection rate of BCG against leprosy was approximately 40% over 30 years (85). In addition, an observational study evaluated adverse events in patients with NMIBC receiving adjuvant treatment with BCG, mitomycin C (MMC), and chemohyperthermia (86). The results demonstrated no significant clinical differences among BCG, MMC, and chemohyperthermia in patient quality of life and side effects. In contrast, another study found a rapid and marked reduction in bladder volume in one patient after BCG instillation (87). However, a study compared the efficacy of chemotherapeutic drugs and BCG’s efficacy in treating bladder cancer in mice and found that cyclophosphamide significantly reduced tumor volume in mice compared with the control group, but the BCG vaccine did not enhance the anti-tumor effect of cyclophosphamide, and BCG treatment alone promoted tumor growth (88). These results indicate that although BCG has been used to treat NMIBC for decades, its therapeutic effect is still controversial, and a more in-depth understanding of its mechanism is needed (89).

Moreover, infection with M. tb is accompanied by increased circulating Treg cells (90). BCG can be used to treat autoimmune type 1 diabetes mellitus (T1DM) due to its ability to induce immunosuppressive T cells (Tregs) to restore the immune balance, and this effect can last for 2-3 years (91). In a Phase I clinical trial, patients with T1DM who received at least two doses of the BCG vaccine could return to normal glucose levels after three years, and similar treatment effects were observed even in patients with advanced diabetes for more than 20 years (92). Interestingly, the therapeutic effect of BCG can last for more than five years (92). In addition, one study found that postnatal BCG vaccination was protective in females with T1DM but not in those who did not receive BCG (93). The underlying mechanisms of BCG vaccination on T1DM may be: 1) BCG vaccination up-regulates Myc, activates nearly 24 Myc target genes under four metabolic pathways, and finally improves glucose metabolism in T1DM patients (94); 2) BCG vaccination induces an increase in the methylation levels of H3K4me3 (Histone 3 Lysine 4) and H3K36me3 (Histone 3 Lysine 36me2), which activates cytokines (TNF, IL-6, and TLR4) related to BCG therapy in T1DM patients (95). These findings suggest that BCG treatment of T1DM improves glycemic control by altering metabolism and persistence. However, a systematic review and meta-analysis revealed that although there was a trend toward improvement in glycated hemoglobin levels after BCG treatment, there was still a lack of strong evidence to support BCG’s efficacy in treating T1DM (96).

Besides, BCG can regulate the progression of atherosclerosis. For example, one study evaluated the effect of subcutaneous BCG vaccination on the development of atherosclerosis in ApoE ^−/−^ mice. It was found that BCG vaccination reduced plaque number and macrophage content but increased lipid content in a mouse model of atherosclerosis (97). Similarly, in another animal study, the treatment of BCG killed by extended freeze-drying significantly reduced the size of atherosclerotic lesions, upregulated IL-10 production, and downregulated the expression of pro-inflammatory cytokines (IL-6, IL-13, and TNF-α) in mice (98). Furthermore, BCG can reduce plasma total cholesterol levels in mice in the experimental group, reduce foam cell formation in peritoneal macrophages, delay the progression of atherosclerotic lesions at the root of the aortic, and decrease the severity of lesions (99).

## 5 The effect of BCG on coronavirus infections

### 5.1 Amino acid sequence similarity between HSP65 protein of BCG strain and spike or nuclear protein of SARS-CoV-2

In the past 20 years, coronavirus (CoV) has triggered three major epidemic outbreaks worldwide, including severe acute respiratory syndrome (SARS), Middle East respiratory syndrome (MERS), and coronavirus disease 2019 (COVID-19) (100). On January 30, 2020, the WHO declared the severe acute respiratory syndrome coronavirus 2 (SARS-CoV-2) outbreak as a public health emergency of international concern. On February 11, 2020, the WHO officially named the pandemic as COVID-19 (101). SARS-CoV-2 is a new type of β coronavirus with the characteristics typical of the coronavirus family (102). Although SARS-CoV-2 infection does not cause as high a fatality rate as SARS-CoV or MERS-CoV, it is more transmissible (100). HSP 65, a major immunogenic component in BCG, is responsible for inducing efficient antigen-specific cell activation (103). A previous study comprehensively compared and analyzed the sequence similarity between the HSP65 protein of BCG strain and the spike and nuclear proteins of SARS-CoV-2, and the results show that the HSP65 protein sequence has a high similarity with the sequence of the spike and nuclear proteins of SARS-CoV-2 (104). This fundamental similarity suggests that BCG can induce cross-immune responses with SARS-CoV-2 antigens to reduce the susceptibility and severity of SARS-CoV-2 infection.

Additionally, studies have shown that BCG contains amino acid sequences similar to SARS-CoV-2 and has moderate to a high binding affinity for multiple common the human leukocyte antigen (HLA), suggesting that BCG vaccination can produce cross-reactive T cells against SARS-CoV-2 (105). HLA class I molecules typically bind peptides (mainly nine amino acids) in the length of 8-11 amino acids. Therefore, one study utilized two computer algorithms to analyze similar 9-amino acid sequences between SARS-CoV-2 and Mycobacterium bovis. The results showed that there were six distinct but closely related groups of peptides between SARS-CoV-2 and *Mycobacterium bovis*, and *M. bovis* contained many seven amino acid sequences that were identical to SARS-CoV-2 (106). Two computer algorithms analyzed the amino acid sequences of SARS-CoV-2 and *M. bovis*, and analysis using IEDB showed that six of the seven distinct but closely related peptides between SARS-CoV-2 and Mycobacterium bovis had moderate to a high binding affinity for multiple common HLA class I molecules. Analysis using NetMHCpan 4.1 showed that four groups of these similar peptides had weak to high binding affinities for common HLA class I molecules. HLA-binding affinity analysis in the computer showed that these very similar 9-mer peptides could be T-cell epitopes. BCG’s similar amino acid sequence to SARS-CoV-2 has the potential to induce cross-reactive T cells against SARS-CoV-2.

### 5.2 The potential protective role of BCG in fighting against COVID-19

#### 5.2.1 COVID-19 Vaccine and its challenges

As of 29 November 2022, a total of 13,042,112,489 COVID-19 vaccine doses have been administered (https://covid19.who.int/). Currently, 199 COVID-19-specific vaccines are evaluated in animal models, and 175 COVID-19-specific vaccines are assessed in clinical trials (https://www.who.int/publications/m/item/draft-landscape-of-covid-19-candidate-vaccines). COVID-19 vaccines approved for emergency use can be grouped into four categories: inactivated vaccines, subunit vaccines, mRNA vaccines, and viral vector-based vaccines. A meta-analysis found that the overall effectiveness of inactivated vaccine, subunit vaccine, mRNA vaccine, and viral vector vaccine against SARS-CoV-2 was 73.11%, 89.33%, 94.29%, and 79.56%, respectively (107). Although these COVID-19 vaccines have shown excellent performance in protecting individuals from SARS-CoV-2 infection, there are concerns about their adverse effects in the population. A previous study has indicated that joint pain, muscle pain, and fever were common adverse events, about 50% of people experience these conditions after receiving the COVID-19 vaccine (107). Other common complications are headaches, chills, and fatigue (22, 108–112). In addition to these common adverse events, rare and severe adverse events have also been reported. For example, myocarditis or pericarditis and thromboembolism cases were reported in people who received viral vector and mRNA vaccines, and such adverse events might cause death (113–116).

In addition, acceptance is also one of the essential factors affecting the global distribution of COVID-19 vaccines. As early as 2021, Jeffrey V Lazarus and colleagues conducted a global survey of potential acceptance of a COVID-19 vaccine among 13,426 people in 19 countries (117). It was found that 71.5% of the participants were willing to receive COVID-19 vaccines, but up to 48.1% of the participants had concerns about the safety of COVID-19 vaccines (117). It is worrisome that COVID-19 vaccine acceptance is not only low among the general population but also among healthcare workers (HCWs). A systematic review and meta-analysis involving 23,739 African HCWs found that COVID-19 vaccine acceptance among African HCWs was only 56.59 (95%CI; 46.26-66.92; I2 = 99.6%, p = 0.000) (118).

The low COVID-19 vaccine acceptance reflects people’s hesitations and refusal to receive COVID-19 vaccines (119). As mentioned above, most of the new COVID-19 vaccines are still in preclinical studies or clinical trials, and the evidence of their effect and complications is unclear. According to a survey, one of the clearest reasons for refusing to be vaccinated is that a vaccine developed in a hurry is considered dangerous, and its efficacy and safety have not been confirmed (120). In addition, people with a medical background are generally expected to be more receptive to COVID-19 vaccines, but a European study suggests contrary results, showing no significant differences in COVID-19 vaccine acceptance between HCWs and non-HCWs (121). Unlike vaccine hesitance faced by COVID-19-specific vaccines, BCG has been used to prevent tuberculosis for more than 100 years, and its safety is widely recognized. Once BCG has been shown to be useful for COVID-19 prevention, it could be adopted by many more people.

#### 5.2.2 The potential of BCG to prevent COVID-19

Based on BCG-induced trained immunity and the advantages of BCG mentioned above, a hypothesis has been proposed that BCG vaccination may reduce the morbidity and mortality of patients with COVID-19. Previous studies have shown that in countries without a universal BCG vaccination, such as Italy, the Netherlands, and the United States, populations were more likely to be infected by COVID-19 than in countries with universal and long-term BCG vaccination and countries that introduced BCG vaccine late to young children, such as Iran, have reported higher death rates from COVID-19 (122). Furthermore, it has been reported that BCG vaccination may reduce viremia after SARS-CoV-2 exposure, decrease the severity of COVID-19, and recover more quickly (123). Interestingly, BCG is a suitable vector for expressing the SARS-CoV-2 antigens. Furthermore, the trained immunity induced by the recombinant BCG vaccine and the SARS-CoV-2 specific immune response could cause a robust protective effect against COVID-19 (124). A study analyzed the association between BCG vaccination coverage and the incidence and mortality of COVID-19 (11). This study divided the countries into three groups according to their BCG coverage (coverage ≥90%, BCG has been recommended but coverage <90%, and BCG has never been introduced). The result showed that countries with higher BCG coverage showed a significantly lower incidence of COVID-19, and the mortality was much lower in countries with BCG coverage ≥90% (11). However, it is essential to note that these data are based on epidemiological and statistical analyses, and can be confounded by many factors, such as race, region, medical and economic level, weather changes, and different stages of COVID-19 (13). Therefore, a study that collected data in 171 countries and adjusted socioeconomic and weather changes showed a 30-fold reduction in COVID-19 mortality in countries with universal BCG vaccination compared to countries without universal BCG vaccination (125).

#### 5.2.3 Landscape of clinical trials evaluating BCG vaccination against COVID-19

In recent years, a growing number of clinical trials have been conducted to evaluate the BCG vaccine’s effectiveness in preventing COVID-19 (**Table 3**). According to the data on ClinicalTrials.gov (https://clinicaltrials.gov/), 31 clinical trials have been conducted to investigate the immune effects of the BCG vaccine on COVID-19. We found that these clinical trials mainly focused on HCWs and older adults (≥60 years old) at high risk of COVID-19. Although some evidence has suggested that the BCG vaccine can beneficially influence the incidence and severity of COVID-19, the study of the long-term effects of BCG on COVID-19 are insufficient. A clinical trial, a 5-year cohort study, in older adults was conducted by Radbond University Medical Center to explore the long-term effects of BCG for infectious and inflammatory diseases, including COVID-19 (NCT05387655). Additionally, as mentioned before, the BCG vaccine has non-specific effects on the immune system, and WHO did not recommend BCG revaccination. Therefore, a clinical trial (NCT04347876) aimed to demonstrate how the previous BCG vaccination could protect people against infection with COVID-19, which mainly focused on altering the prognosis of COVID-19. Similarly, another clinical trial (NCT04369794) was developed to evaluate the impact of previous BCG vaccination and BCG revaccination on SARS-CoV-2 at different phases and disease phenotypes. These clinical trials focused on assessing the effect of BCG on reducing the incidence and severity of COVID-19. In contrast, an early Phase 1 clinical trial (NCT02403505) was conducted to evaluate COVID-19 Antigen Presentation Therapeutic Biological Product Mis for treating multiple gene mutation COVID-19 virus strains and activating human COVID-10 antigen presentation reaction. This clinical trial will recruit 20 participants with COVID-19, and the intervention was Ad26 COVID-19 Spike 1.0mL plus TICE^®^BCG Organism 50mg.

**Table 3:** Clinical Trials of BCG fighting against COVID-19.

Trial ID	Countries	Status	Sample size	Intervention	Primary outcome
NCT05387655	Netherlands	Enrolling by invitation	500 older people	Case Control, BCG and Placebo	Incidence of infectious and inflammatory disease
NCT04347876	Egypt	Unknown	100 participants 12–80 years old	Case Control (Observational)	Pneumonia severity index and need for ICU admission
NCT04648800	Poland	Recruiting Phase 3	1000 participants, 25 years and older	Interventional clinical trial, BCG-10 vaccine and 0.9% saline	Death and life- or health-threatening condition
NCT04369794	Brazil	Active, not recruiting Phase 4	400 participants	Dose of 0.1 mL of BCG (2 and 8 x 1.000.000 CFU) and 0.1 ml of 0.9% NaCl saline solution	1. Clinical evolution of COVID-19, classified as mild, moderate and severe 2. SARS-CoV-2 elimination, virus detection by PCR 3.Seroconversion rate and titration, titration of anti SARS-CoV-2 IgA, IgM and IgG
NCT04659941	Brazil	Active, not recruiting Phase 2	753 participants	Dose of 0.1 mL of BCG and 0.1 ml of 0.9% NaCl saline solution	1. Compare the cumulative incidence of SARS-CoV-2 infection. 2. Compare the cumulative incidence of severe forms of COVID-19. 3. Assess the BCG vaccine-mediated immune response in health care workers.
NCT04379336	South Africa	Complete Phase 3	1000 adults	BCG and 0.9% Sodium Chloride	Incidence of HCWs hospitalized due to COVID-19 per arm
NCT04641858	Denmark	Active, not recruiting Phase 4	668 adults	BCG-Denmark and 0.9% Sodium Chloride	Days of unplanned absenteeism due to illness, unplanned absenteeism is defined by being absent from work due to causes other than holidays, parental leave, and other planned leaves, family assistance (including mourning leave) and quarantine measures.
NCT04327206	Australia	Complete Phase 3	6828 adults	BCG (Danish strain 1331) and 0.9% Sodium Chloride	Symptomatic COVID-19 by 6 months Severe COVID-19 incidence over 6 months
NCT04461379	Mexico	Active, not recruiting Phase 3	908 adults	BCG (Tokio 172 strain) and 0.9% Sodium Chloride	1. Demonstrate COVID- 19 disease incidence among Health care workers 2. Demonstrate cumulative incidence of hospitalization for COVID-19 among Health care workers 3. Demonstrate the Incidence of specific Antibodies against SARS-CoV-2 at 3 and 6 months in health care workers 4. Hospitalization of severe disease COVID-19 5. Oxygen supplementation in severe disease COVID-19 6. Need for intubation or non-invasive ventilation for the patient 7. Critical care admission with SARS-CoV-2 8. Mortality associated to progressive pulmonary disease
NCT04373291	Denmark	Complete Phase 3	1293 adults	BCG-Denmark and 0.9% Sodium Chloride	Number of days of unplanned absenteeism for any reason
NCT04648800	Poland	Recruiting Phase 3	1000 adults	BCG-10 vaccine and 0.9% Sodium Chloride	Death and life- or health-threatening condition
NCT04537663	Netherlands	Recruiting Phase 4	5200 Elderly	BCG (Danish strain) and 0.9% Sodium Chloride	The trial has an adaptive primary endpoint. Based on predefined objective and quantitative criteria the primary endpoint will be either a clinically relevant respiratory tract infection, or COVID-19
NCT04384614	Tunisia	Withdraw	N/A	Cross-sectional	1. Differences related to epidemiological demographic characteristics 2. Biospecimen Retention: Samples With DNA
NCT04475302	India	Complete Phase 3	2175 Elderly	BCG	Mortality due to COVID-19 disease
NCT04384549	France	Unknown Phase 3	1120 participants	BCG vaccine (AJ Vaccine) and 0.9% Sodium Chloride	Incidence of documented COVID-19 among health care workers exposed to SARS- CoV-2 and vaccinated with BCG compared to placebo.
NCT04542330	Denmark	Active, not recruiting Phase 3	1700 Elderly (65-110 years old)	BCG-Denmark and 0.9% Sodium Chloride	Acute infection, acute infection identified either by a doctor, antibiotics use, hospitalization or death due to infection.
NCT04414267	Greece	Complete Phase 4	301 Participants (50 Years and older)	BCG and 0.9% Sodium Chloride	Positive for the respiratory questionnaire consisted of questions concerning the appearance of symptoms possibly, probably and/or definitively related to COVID-19 on visit 3
NCT04439045	Canada	Completed Phase 3	122 participants	VPM1002 is a recombinant BCG (rBCG) and 0.9% Sodium Chloride	COVID-19 infection, to compare the self-reported incidence of SARS-CoV-2 infection (confirmed by positive test) following vaccination with either VPM1002 or placebo.
NCT04348370	United States	Active, not recruiting Phase 4	1800 Participants (18 Years to 75 Years)	BCG Vaccine And Placebo Vaccine	Incidence of COVID 19 Infection
NCT02081326	United States	Active, not recruiting Phase 2	150 Participants (18 Years to 65 Years)	BCG and 0.9% Sodium Chloride	1. Number of Type 1 Diabetics with COVID-19 symptomatic infections 2. Impact of COVID-19 (severity, duration of symptoms, absence from work) 3. Reported Rates of Infectious Diseases
NCT04826718	Cape Verde	Enrolling by invitation	400 Participants (18 Years and older)	Observational Questionaire Capillary blood collection Collection of peripheral venous blood	1. Total number of days absent from work due to COVID-19 2. Unplanned Absenteeism 3. Symptomatology after infection by SARS-CoV-2 4. Presence or absence of anti-SARS-CoV-2 Acs 5. Duration of anti-SARS-CoV-2 Acs
NCT04387409	Germany	Active, not recruiting Phase 3	59 Participants (18 Years and older)	VPM1002 is a recombinant BCG (rBCG) and 0.9% Sodium Chloride	Number of days absent from work due to respiratory disease
NCT04435379	Germany	Completed Phase 3	2038 Participants	VPM1002 is a recombinant BCG (rBCG) and 0.9% Sodium	Number of days with severe respiratory disease at hospital and/or at home

However, studies have also shown that BCG vaccination does not significantly protect against COVID-19. In a multicenter, randomized, double-blind, placebo-controlled phase III clinical trial in Poland in which investigators revaccinated HCWs with BCG, the results showed no significant difference in the incidence of COVID-19 between placebo and BCG groups (126). Results from another double-blind, randomized, controlled, phase 3 trial of HCWs in South Africa showed that vaccinating HCWs with BCG did not reduce the risk of COVID-19 and hospitalization for severe COVID-19 (127). In terms of the reasons contrary to other research findings, firstly, the success of BCG vaccination has not been verified. It can be seen that these two studies did not clarify whether TST-negative participants turned positive after receiving BCG, so this does not determine the protective efficacy of BCG. In addition, LTBI should receive significant concern. An animal study showed that Mycobacterium tuberculosis infection inhibited the trained immune processes in the bone marrow. Therefore, LTBI may have negatively influenced the non-specific role of BCG (128). However, these two studies did not address the confounding factor of LTBI.

## 6 Conclusions

The century-old BCG vaccine remains the only vaccine approved for TB prevention. BCG can induce trained immunity against the invasion of pathogens other than *Mycobacterium tuberculosis*, such as respiratory viruses. Based on this theory, it is speculated that BCG may potentially prevent COVID-19 morbidity and mortality. To verify this hypothesis, scientists worldwide have carried out a large number of epidemiological studies and statistical analyses, but the results of these studies are highly heterogeneous due to the interference of a variety of confounding factors. Therefore, more than 30 clinical trials have been conducted to evaluate the immune effect of BCG on COVID-19, and some clinical trials are exploring the long-term immune effects of BCG and the clinical benefits of COVID-19 antigen presentation therapeutic biological product mixed BCG on COVID-19. The protective effect of BCG on newborns and children is obvious, but the immune effect of BCG is not lifelong, and it is a question worth discussing how to produce immune effects on adults, especially adults without antibodies.

## Author contributions

Conceptualization: JD and WG. Methodology: JD, YS, RW, ED, YC, WZ, and WG. Data Analysis: JD and YS. Software: YS and WG. Writing Original Manuscript: JD and YS. Review and revising manuscript: JD and WG. Funding Acquisition: JD and WG. All authors reviewed and approved the final manuscript.

## References

[1] WHO. Global tuberculosis report 2022. In: Geneva: World health organization. Geneva: World Health Organization (2022).

[2] WHO. Global tuberculosis report 2021. In: Geneva pp: World health organization (2021). pp. 1–262.

[3] Hussey G Hawkridge T Hanekom W. Childhood tuberculosis: old and new vaccines. Paediatr Respir Rev (2007) 8:148–54. doi: 10.1016/j.prrv.2007.04.009 17574159

[4] Gong W Mao Y Li Y Qi Y. BCG Vaccination: A potential tool against COVID-19 and COVID-19-like black swan incidents. Int Immunopharmacol (2022) 108:108870. doi: 10.1016/j.intimp.2022.108870 35597119 PMC9113676

[5] Gong W An H Wang J Cheng P Qi Y. The natural effect of BCG vaccination on COVID-19: The debate continues. Front Immunol (2022) 13:953228. doi: 10.3389/fimmu.2022.953228 35898508 PMC9309283

[6] Liu W. Basis for implementing the BCG replanting strategy. Chin J Antituberculosis (1998) 20:3.

[7] Kumar NP Padmapriyadarsini C Rajamanickam A Bhavani PK Nancy A Jeyadeepa B. BCG Vaccination induces enhanced frequencies of dendritic cells and altered plasma levels of type I and type III interferons in elderly individuals. Int J Infect Dis IJID Off Publ Int Soc Infect Dis (2021) 110:98–104. doi: 10.1016/j.ijid.2021.07.041 PMC8295056 34302964

[8] Netea MG Joosten LA Latz E Mills KH Natoli G Stunnenberg HG. Trained immunity: A program of innate immune memory in health and disease. Science (2016) 352:aaf1098. doi: 10.1126/science.aaf1098 27102489 PMC5087274

[9] Kleinnijenhuis J Quintin J Preijers F Joosten LA Ifrim DC Saeed S. Bacille calmette-guerin induces NOD2-dependent nonspecific protection from reinfection *via* epigenetic reprogramming of monocytes. Proc Natl Acad Sci United States America (2012) 109:17537–42. doi: 10.1073/pnas.1202870109 PMC3491454 22988082

[10] Aspatwar A Gong W Wang S Wu X Parkkila S. Tuberculosis vaccine BCG: the magical effect of the old vaccine in the fight against the COVID-19 pandemic. Int Rev Immunol (2022) 41:283–96. doi: 10.1080/08830185.2021.1922685 PMC8108189 33960271

[11] Gong W Wu X. Is the tuberculosis vaccine BCG an alternative weapon for developing countries to defeat COVID-19? Indian J tuberculosis (2021) 68:401–4. doi: 10.1016/j.ijtb.2020.10.012 PMC7641523 34099209

[12] Gong W Aspatwar A Wang S Parkkila S Wu X. COVID-19 pandemic: SARS-CoV-2 specific vaccines and challenges, protection *via* BCG trained immunity, and clinical trials. Expert Rev Vaccines (2021) 20:857–80. doi: 10.1080/14760584.2021.1938550 PMC8220438 34078215

[13] Wang J Zhang Q Wang H Gong W. The potential roles of BCG vaccine in the prevention or treatment of COVID-19. Front Biosci (Landmark Ed) (2022) 27:157. doi: 10.31083/j.fbl2705157 35638424

[14] Ujiie A Watanabe M Ushiba D. Non-specific resistance of the BCG vaccinated mice to the infection with salmonella enteritidis (I). Nihon Saikingaku Zasshi (1966) 21:675–82. doi: 10.3412/jsb.21.675 6010589

[15] Doroftei B Ciobica A Ilie OD Maftei R Ilea C. Mini-review discussing the reliability and efficiency of COVID-19 vaccines. Diagnostics (Basel) (2021) 11:579. doi: 10.3390/diagnostics11040579 33804914 PMC8063839

[16] Jia Z Gong W. Will mutations in the spike protein of SARS-CoV-2 lead to the failure of COVID-19 vaccines? J Korean Med Sci (2021) 36:e124. doi: 10.3346/jkms.2021.36.e124 33975397 PMC8111046

[17] Qin S Cui M Sun S Zhou J Du Z Cui Y. Genome characterization and potential risk assessment of the novel SARS-CoV-2 variant omicron (B.1.1.529). Zoonoses (2021) 1:18. doi: 10.15212/ZOONOSES-2021-0024

[18] Garcia-Beltran WF St Denis KJ Hoelzemer A Lam EC Nitido AD Sheehan ML. mRNA-based COVID-19 vaccine boosters induce neutralizing immunity against SARS-CoV-2 omicron variant. Cell (2022) 185:457–66.e4. doi: 10.1016/j.cell.2021.12.033 34995482 PMC8733787

[19] Wang H Zhang Y Huang B Deng W Quan Y Wang W. Development of an inactivated vaccine candidate, BBIBP-CorV, with potent protection against SARS-CoV-2. Cell (2020) 182:713–21 e9. doi: 10.1016/j.cell.2020.06.008 32778225 PMC7275151

[20] Huang B Dai L Wang H Hu Z Yang X Tan W. Serum sample neutralisation of BBIBP-CorV and ZF2001 vaccines to SARS-CoV-2 501Y.V2. Lancet Microbe (2021) 2:e285. doi: 10.1016/S2666-5247(21)00082-3 33870240 PMC8043583

[21] Dolgin E. CureVac COVID vaccine let-down spotlights mRNA design challenges. Nature (2021) 594:483. doi: 10.1038/d41586-021-01661-0 34145413

[22] Polack FP Thomas SJ Kitchin N Absalon J Gurtman A Lockhart S. Safety and efficacy of the BNT162b2 mRNA covid-19 vaccine. New Engl J Med (2020) 383:2603–15. doi: 10.1056/NEJMoa2034577 PMC7745181 33301246

[23] Wu S Huang J Zhang Z Wu J Zhang J Hu H. Safety, tolerability, and immunogenicity of an aerosolised adenovirus type-5 vector-based COVID-19 vaccine (Ad5-nCoV) in adults: preliminary report of an open-label and randomised phase 1 clinical trial. Lancet Infect Dis (2021) 21:1654–64. doi: 10.1016/S1473-3099(21)00396-0 PMC8313090 34324836

[24] Stephenson KE Le Gars M Sadoff J de Groot AM Heerwegh D Truyers C. Immunogenicity of the Ad26.COV2.S vaccine for COVID-19. JAMA (2021) 325:1535–44. doi: 10.1001/jama.2021.3645 PMC7953339 33704352

[25] Emary KRW Golubchik T Aley PK Ariani CV Angus B Bibi S. Efficacy of ChAdOx1 nCoV-19 (AZD1222) vaccine against SARS-CoV-2 variant of concern 202012/01 (B.1.1.7): an exploratory analysis of a randomised controlled trial. Lancet (2021) 397:1351–62. doi: 10.1016/S0140-6736(21)00628-0 PMC8009612 33798499

[26] Ella R Reddy S Blackwelder W Potdar V Yadav P Sarangi V. Efficacy, safety, and lot-to-lot immunogenicity of an inactivated SARS-CoV-2 vaccine (BBV152): interim results of a randomised, double-blind, controlled, phase 3 trial. Lancet (2021) 398:2173–84. doi: 10.1016/S0140-6736(21)02000-6 PMC8584828 34774196

[27] Gong W Parkkila S Wu X Aspatwar A. SARS-CoV-2 variants and COVID-19 vaccines: Current challenges and future strategies. Int Rev Immunol (2022) 41:1–22. doi: 10.1080/08830185.2022.2079642 35635216

[28] Czajka H Zapolnik P Krzych Ł Kmiecik W Stopyra L Nowakowska A. A multi-center, randomised, double-blind, placebo-controlled phase III clinical trial evaluating the impact of BCG re-vaccination on the incidence and severity of SARS-CoV-2 infections among symptomatic healthcare professionals during the COVID-19 pandemic in Poland–first results. Vaccines (2022) 10:314. doi: 10.3390/vaccines10020314 35214772 PMC8879775

[29] Singh S Khera D Chugh A Khasbage S Khera PS Chugh VK. BCG Vaccination impact on mortality and recovery rates in COVID-19: A meta-analysis. Monaldi Arch Chest Dis (2021) 91. doi: 10.4081/monaldi.2021.1875 34461704

[30] XM Z CH X XW P F Z. Progress in researches on novel tuberculosis vaccine for different populations:a review. Chin J Publ Heal (2021) 11:1698–703. doi: 10.11847/zgggws1133162

[31] Ren L Di LY. New understanding and prospects for tuberculosis vaccines. Chin Med J Metallurgical Industry (2016) 33:143.

[32] Mulder WJM Ochando J Joosten LAB Fayad ZA Netea MG. Therapeutic targeting of trained immunity. Nat Rev Drug Discov (2019) 18:553–66. doi: 10.1038/s41573-019-0025-4 PMC7069501 30967658

[33] Netea MG Domínguez-Andrés J Barreiro LB Chavakis T Divangahi M Fuchs E. Defining trained immunity and its role in health and disease. Nat Rev Immunol (2020) 20:375–88. doi: 10.1038/s41577-020-0285-6 PMC7186935 32132681

[34] Roquilly A Jacqueline C Davieau M Mollé A Sadek A Fourgeux C. Alveolar macrophages are epigenetically altered after inflammation, leading to long-term lung immunoparalysis. Nat Immunol (2020) 21:636–48. doi: 10.1038/s41590-020-0673-x 32424365

[35] Trunz BB Fine P Dye C. Effect of BCG vaccination on childhood tuberculous meningitis and miliary tuberculosis worldwide: a meta-analysis and assessment of cost-effectiveness. Lancet (2006) 367:1173–80. doi: 10.1016/S0140-6736(06)68507-3 16616560

[36] Verma D Chan ED Ordway DJ. Non-tuberculous mycobacteria interference with BCG-current controversies and future directions. Vaccines (Basel) (2020) 8:688. doi: 10.3390/vaccines8040688 33207695 PMC7711602

[37] Kaveh DA Garcia-Pelayo MC Hogarth PJ. Persistent BCG bacilli perpetuate CD4 T effector memory and optimal protection against tuberculosis. Vaccine (2014) 32:6911–8. doi: 10.1016/j.vaccine.2014.10.041 25444816

[38] Moliva JI Turner J Torrelles JB. Prospects in mycobacterium bovis bacille calmette et guérin (BCG) vaccine diversity and delivery: why does BCG fail to protect against tuberculosis? Vaccine (2015) 33:5035–41. doi: 10.1016/j.vaccine.2015.08.033 PMC4577463 26319069

[39] Gela A Murphy M Rodo M Hadley K Hanekom WA Boom WH. Effects of BCG vaccination on donor unrestricted T cells in two prospective cohort studies. EBioMedicine (2022) 76:103839. doi: 10.1016/j.ebiom.2022.103839 35149285 PMC8842032

[40] Sutiwisesak R Hicks ND Boyce S Murphy KC Papavinasasundaram K Carpenter SM. A natural polymorphism of mycobacterium tuberculosis in the esxH gene disrupts immunodomination by the TB10.4-specific CD8 T cell response. PloS Pathog (2020) 16:e1009000. doi: 10.1371/journal.ppat.1009000 33075106 PMC7597557

[41] Moreira-Teixeira L Stimpson PJ Stavropoulos E Hadebe S Chakravarty P Ioannou M. Type I IFN exacerbates disease in tuberculosis-susceptible mice by inducing neutrophil-mediated lung inflammation and NETosis. Nat Commun (2020) 11:5566. doi: 10.1038/s41467-020-19412-6 33149141 PMC7643080

[42] Chen T Blanc C Eder AZ Prados-Rosales R Souza AC Kim RS. Association of human antibodies to arabinomannan with enhanced mycobacterial opsonophagocytosis and intracellular growth reduction. J Infect Dis (2016) 214:300–10. doi: 10.1093/infdis/jiw141 PMC4918826 27056953

[43] Zhou KL Li X Zhang XL Pan Q. Mycobacterial mannose-capped lipoarabinomannan: a modulator bridging innate and adaptive immunity. Emerg Microbes Infect (2019) 8:1168–77. doi: 10.1080/22221751.2019.1649097 PMC6713153 31379262

[44] Beveridge NE Price DA Casazza JP Pathan AA Sander CR Asher TE. Immunisation with BCG and recombinant MVA85A induces long-lasting, polyfunctional mycobacterium tuberculosis-specific CD4+ memory T lymphocyte populations. Eur J Immunol (2007) 37:3089–100. doi: 10.1002/eji.200737504 PMC2365909 17948267

[45] Soto JA Gálvez NMS Andrade CA Ramírez MA Riedel CA Kalergis AM. BCG Vaccination induces cross-protective immunity against pathogenic microorganisms. Trends Immunol (2022) 43:322–35. doi: 10.1016/j.it.2021.12.006 35074254

[46] Jeyanathan M Vaseghi-Shanjani M Afkhami S Grondin JA Kang A D'Agostino MR. Parenteral BCG vaccine induces lung-resident memory macrophages and trained immunity *via* the gut-lung axis. Nat Immunol (2022) 23:1687–702. doi: 10.1038/s41590-022-01354-4 PMC9747617 36456739

[47] Arts RJW Carvalho A La Rocca C Palma C Rodrigues F Silvestre R. Immunometabolic pathways in BCG-induced trained immunity. Cell Rep (2016) 17:2562–71. doi: 10.1016/j.celrep.2016.11.011 PMC5177620 27926861

[48] Bickett TE McLean J Creissen E Izzo L Hagan C Izzo AJ. Characterizing the BCG induced macrophage and neutrophil mechanisms for defense against mycobacterium tuberculosis. Front Immunol (2020) 11:1202. doi: 10.3389/fimmu.2020.01202 32625209 PMC7314953

[49] Grassin-Delyle S Abrial C Salvator H Brollo M Naline E Devillier P. The role of toll-like receptors in the production of cytokines by human lung macrophages. J Innate Immun (2020) 12:63–73. doi: 10.1159/000494463 30557876 PMC6959095

[50] Wannigama DL Jacquet A. NOD2-dependent BCG-induced trained immunity: A way to regulate innate responses to SARS-CoV2? Int J Infect Dis IJID Off Publ Int Soc Infect Dis (2020) 101:52–5. doi: 10.1016/j.ijid.2020.09.1429 PMC7832069 32980554

[51] Ferluga J Yasmin H Al-Ahdal MN Bhakta S Kishore U. Natural and trained innate immunity against mycobacterium tuberculosis. Immunobiology (2020) 225:151951. doi: 10.1016/j.imbio.2020.151951 32423788

[52] Joosten SA van Meijgaarden KE Arend SM Prins C Oftung F Korsvold GE. Mycobacterial growth inhibition is associated with trained innate immunity. J Clin Invest (2018) 128:1837–51. doi: 10.1172/JCI97508 PMC5919803 29461976

[53] Iqbal NT Hussain R. Non-specific immunity of BCG vaccine: A perspective of BCG immunotherapy. Trials Vaccinol (2014) 3:143–9. doi: 10.1016/j.trivac.2014.08.002

[54] Liu Y Liang S Ding R Hou Y Deng F Ma X. BCG-Induced trained immunity in macrophage: reprograming of glucose metabolism. Int Rev Immunol (2020) 39:83–96. doi: 10.1080/08830185.2020.1712379 31933415

[55] Funes SC Rios M Escobar-Vera J Kalergis AM. Implications of macrophage polarization in autoimmunity. Immunology (2018) 154:186–95. doi: 10.1111/imm.12910 PMC5980179 29455468

[56] Boutilier AJ Elsawa SF. Macrophage polarization states in the tumor microenvironment. Int J Mol Sci (2021) 22:6995. doi: 10.3390/ijms22136995 34209703 PMC8268869

[57] Misson P van den Brûle S Barbarin V Lison D Huaux F. Markers of macrophage differentiation in experimental silicosis. J Leukoc Biol (2004) 76:926–32. doi: 10.1189/jlb.0104019 15292275

[58] Shapouri-Moghaddam A Mohammadian S Vazini H Taghadosi M Esmaeili SA Mardani F. Macrophage plasticity, polarization, and function in health and disease. J Cell Physiol (2018) 233:6425–40. doi: 10.1002/jcp.26429 PMC13482560 29319160

[59] Dos Santos CC Walburg KV van Veen S Wilson LG Trufen CEM Nascimento IP. Recombinant BCG-LTAK63 vaccine candidate for tuberculosis induces an inflammatory profile in human macrophages. Vaccines (Basel) (2022) 10:831. doi: 10.3390/vaccines10060831 35746439 PMC9227035

[60] Mills CD Kincaid K Alt JM Heilman MJ Hill AM. M-1/M-2 macrophages and the Th1/Th2 paradigm. J Immunol (Baltimore Md 1950) (2000) 164:6166–73. doi: 10.4049/jimmunol.164.12.6166 10843666

[61] Zhang X Mosser DM. Macrophage activation by endogenous danger signals. J Pathol (2008) 214:161–78. doi: 10.1002/path.2284 PMC2724989 18161744

[62] Wang T Liu HY Lian G Zhang SY Wang X Jiang CT. HIF1 alpha-induced glycolysis metabolism is essential to the activation of inflammatory macrophages. Mediators Inflammation (2017) 2017:10. doi: 10.1155/2017/9029327 PMC5745720 29386753

[63] Liu Q Tian Y Zhao X Jing H Xie Q Li P. NMAAP1 expressed in BCG-activated macrophage promotes M1 macrophage polarization. Molecules Cells (2015) 38:886–94. doi: 10.14348/molcells.2015.0125 PMC4625070 26429502

[64] Debisarun PA Kilic G de Bree LCJ Pennings LJ van Ingen J Benn CS. The impact of BCG dose and revaccination on trained immunity. Clin Immunol (2023) 246:109208. doi: 10.1016/j.clim.2022.109208 36565972

[65] Dye C. Making wider use of the world's most widely used vaccine: Bacille calmette–guérin revaccination reconsidered. J R Soc Interface (2013) 10:20130365. doi: 10.1098/rsif.2013.0365 23904584 PMC3757998

[66] Rakshit S Ahmed A Adiga V Sundararaj BK Sahoo PN Kenneth J. BCG Revaccination boosts adaptive polyfunctional Th1/Th17 and innate effectors in IGRA+ and IGRA- Indian adults. JCI Insight (2019) 4:e130540. doi: 10.1172/jci.insight.130540 31743110 PMC6975271

[67] Nemes E Geldenhuys H Rozot V Rutkowski KT Ratangee F Bilek N. Prevention of m. tuberculosis infection with H4:IC31 vaccine or BCG revaccination. New Engl J Med (2018) 379:138–49. doi: 10.1056/NEJMoa1714021 PMC5937161 29996082

[68] Suliman S Geldenhuys H Johnson JL Hughes JE Smit E Murphy M. Bacillus calmette-guérin (BCG) revaccination of adults with latent mycobacterium tuberculosis infection induces long-lived BCG-reactive NK cell responses. J Immunol (Baltimore Md 1950) (2016) 197:1100–10. doi: 10.4049/jimmunol.1501996 PMC4976036 27412415

[69] Bekker LG Dintwe O Fiore-Gartland A Middelkoop K Hutter J Williams A. A phase 1b randomized study of the safety and immunological responses to vaccination with H4:IC31, H56:IC31, and BCG revaccination in mycobacterium tuberculosis-uninfected adolescents in cape town, south Africa. EClinicalMedicine (2020) 21:100313. doi: 10.1016/j.eclinm.2020.100313 32382714 PMC7201034

[70] Glynn JR Dube A Fielding K Crampin AC Karonga Prevention Trial G Kanjala C. The effect of BCG revaccination on all-cause mortality beyond infancy: 30-year follow-up of a population-based, double-blind, randomised placebo-controlled trial in Malawi. Lancet Infect Dis (2021) 21:1590–7. doi: 10.1016/S1473-3099(20)30994-4 PMC8550897 34237262

[71] Sadoff J De Paepe E DeVincenzo J Gymnopoulou E Menten J Murray B. Prevention of respiratory syncytial virus infection in healthy adults by a single immunization of Ad26.RSV.preF in a human challenge study. J Infect Dis (2022) 226:396–406. doi: 10.1093/infdis/jiab003 PMC9417128 33400792

[72] Pastula ST Hackett J Coalson J Jiang X Villafana T Ambrose C. Hospitalizations for respiratory syncytial virus among adults in the united states, 1997-2012. Open Forum Infect Dis (2017) 4:ofw270. doi: 10.1093/ofid/ofw270 28480262 PMC5414053

[73] Ackerson B Tseng HF Sy LS Solano Z Slezak J Luo Y. Severe morbidity and mortality associated with respiratory syncytial virus versus influenza infection in hospitalized older adults. Clin Infect Dis an Off Publ Infect Dis Soc America (2019) 69:197–203. doi: 10.1093/cid/ciy991 PMC6603263 30452608

[74] Cespedes PF Rey-Jurado E Espinoza JA Rivera CA Canedo-Marroquin G Bueno SM. A single, low dose of a cGMP recombinant BCG vaccine elicits protective T cell immunity against the human respiratory syncytial virus infection and prevents lung pathology in mice. Vaccine (2017) 35:757–66. doi: 10.1016/j.vaccine.2016.12.048 28065474

[75] Rey-Jurado E Soto J Galvez N Kalergis AM. A safe and efficient BCG vectored vaccine to prevent the disease caused by the human respiratory syncytial virus. Hum Vaccin Immunother (2017) 13:2092–7. doi: 10.1080/21645515.2017.1334026 PMC5612508 28598702

[76] Leentjens J Kox M Stokman R Gerretsen J Diavatopoulos DA van Crevel R. BCG Vaccination enhances the immunogenicity of subsequent influenza vaccination in healthy volunteers: A randomized, placebo-controlled pilot study. J Infect Dis (2015) 212:1930–8. doi: 10.1093/infdis/jiv332 26071565

[77] Mukherjee S Subramaniam R Chen H Smith A Keshava S Shams H. Boosting efferocytosis in alveolar space using BCG vaccine to protect host against influenza pneumonia. PloS One (2017) 12:e0180143. doi: 10.1371/journal.pone.0180143 28686604 PMC5501455

[78] Giamarellos-Bourboulis EJ Tsilika M Moorlag S Antonakos N Kotsaki A Domínguez-Andrés J. Activate: Randomized clinical trial of BCG vaccination against infection in the elderly. Cell (2020) 183:315–23.e9. doi: 10.1016/j.cell.2020.08.051 32941801 PMC7462457

[79] Salem A Nofal A Hosny D. Treatment of common and plane warts in children with topical viable bacillus calmette-guerin. Pediatr Dermatol (2013) 30:60–3. doi: 10.1111/j.1525-1470.2012.01848.x 22958215

[80] Podder I Bhattacharya S Mishra V Sarkar TK Chandra S Sil A. Immunotherapy in viral warts with intradermal bacillus calmette-guerin vaccine versus intradermal tuberculin purified protein derivative: A double-blind, randomized controlled trial comparing effectiveness and safety in a tertiary care center in Eastern India. Indian J Dermatol Venereol Leprol (2017) 83:411. doi: 10.4103/0378-6323.193623 27852999

[81] Moorlag SJCFM Arts RJW van Crevel R Netea MG. Non-specific effects of BCG vaccine on viral infections. Clin Microbiol Infect (2019) 25:1473–8. doi: 10.1016/j.cmi.2019.04.020 31055165

[82] Aaby P Roth A Ravn H Napirna BM Rodrigues A Lisse IM. Randomized trial of BCG vaccination at birth to low-Birth-Weight children: Beneficial nonspecific effects in the neonatal period? J Infect Dis (2011) 204:245–52. doi: 10.1093/infdis/jir240 21673035

[83] Biering-Sørensen S Aaby P Lund N Monteiro I Jensen KJ Eriksen HB. Early BCG-Denmark and neonatal mortality among infants weighing &lt;2500 g: A randomized controlled trial. Clin Infect Dis (2017) 65:1183–90. doi: 10.1093/cid/cix525 PMC5849087 29579158

[84] Bajic P Wolfe AJ Gupta GN. Old instillations and new implications for bladder cancer: the urinary microbiome and intravesical BCG. BJU Int (2019) 124:7–8. doi: 10.1111/bju.14683 30663202

[85] Glynn JR Fielding K Mzembe T Sichali L Banda L McLean E. BCG Re-vaccination in Malawi: 30-year follow-up of a large, randomised, double-blind, placebo-controlled trial. Lancet Global Health (2021) 9:e1451–e9. doi: 10.1016/S2214-109X(21)00309-0 PMC8459381 34534489

[86] González-Padilla DA González-Díaz A Guerrero-Ramos F Rodríguez-Serrano A García-Jarabo E Corona-laPuerta M. Quality of life and adverse events in patients with nonmuscle invasive bladder cancer receiving adjuvant treatment with BCG, MMC, or chemohyperthermia. Urologic Oncol (2021) 39:76.e9–.e14. doi: 10.1016/j.urolonc.2020.07.00 32753359

[87] Yang C Lv Z Li Y. Reduction of bladder volume after BCG immunotherapy. Urologia Internationalis (2021) 105:155–8. doi: 10.1159/000510167 32882690

[88] Soloway MS. Effectiveness of long-term chemotherapy and/or BCG on murine bladder cancer. Natl Cancer Institute monograph (1978) 49:327–32. 748788

[89] Lim CJ Nguyen PHD Wasser M Kumar P Lee YH Nasir NJM. Immunological hallmarks for clinical response to BCG in bladder cancer. Front Immunol (2020) 11:615091. doi: 10.3389/fimmu.2020.615091 33584702 PMC7879685

[90] Cardona P Cardona PJ. Regulatory T cells in mycobacterium tuberculosis infection. Front Immunol (2019) 10:2139. doi: 10.3389/fimmu.2019.02139 31572365 PMC6749097

[91] Keefe RC Takahashi H Tran L Nelson K Ng N Kühtreiber WM. BCG Therapy is associated with long-term, durable induction of treg signature genes by epigenetic modulation. Sci Rep (2021) 11:14933. doi: 10.1038/s41598-021-94529-2 34294806 PMC8298580

[92] Kuhtreiber WM Faustman DL. BCG Therapy for type 1 diabetes: Restoration of balanced immunity and metabolism. Trends Endocrinol Metab (2019) 30:80–92. doi: 10.1016/j.tem.2018.11.006 30600132

[93] Klein BY. Newborn BCG vaccination complemented by boosting correlates better with reduced juvenile diabetes in females, than vaccination alone. Vaccine (2020) 38:6427–34. doi: 10.1016/j.vaccine.2020.07.066 32773242

[94] Kühtreiber WM Takahashi H Keefe RC Song Y Tran L Luck TG. BCG Vaccinations upregulate myc, a central switch for improved glucose metabolism in diabetes. iScience (2020) 23:101085. doi: 10.1016/j.isci.2020.101085 32380424 PMC7205768

[95] Dias HF Kühtreiber WM Nelson KJ Ng NC Zheng H Faustman DL. Epigenetic changes related to glucose metabolism in type 1 diabetes after BCG vaccinations: A vital role for KDM2B. Vaccine (2022) 40:1540–54. doi: 10.1016/j.vaccine.2021.04.011 33933315

[96] Chang YC Lin CJ Hsiao YH Chang YH Liu SJ Hsu HY. Therapeutic effects of BCG vaccination on type 1 diabetes mellitus: A systematic review and meta-analysis of randomized controlled trials. J Diabetes Res (2020) 2020:8954125. doi: 10.1155/2020/8954125 32309449 PMC7139880

[97] Bekkering S Singh K Lu H Limawan AP Nold-Petry CA Wallace MJ. Neonatal subcutaneous BCG vaccination decreases atherosclerotic plaque number and plaque macrophage content in ApoE(-/-) mice. Biology (2022) 11:151. doi: 10.3390/biology11101511 36290415 PMC9599032

[98] Ovchinnikova OA Berge N Kang C Urien C Ketelhuth DF Pottier J. Mycobacterium bovis BCG killed by extended freeze-drying induces an immunoregulatory profile and protects against atherosclerosis. J Intern Med (2014) 275:49–58. doi: 10.1111/joim.12127 23962000

[99] van Dam AD Bekkering S Crasborn M van Beek L van den Berg SM Vrieling F. BCG Lowers plasma cholesterol levels and delays atherosclerotic lesion progression in mice. Atherosclerosis (2016) 251:6–14. doi: 10.1016/j.atherosclerosis.2016.05.031 27232458

[100] Harrison AG Lin T Wang P. Mechanisms of SARS-CoV-2 transmission and pathogenesis. Trends Immunol (2020) 41:1100–15. doi: 10.1016/j.it.2020.10.004 PMC7556779 33132005

[101] Sun P Lu X Xu C Sun W Pan B. Understanding of COVID-19 based on current evidence. J Med virology. (2020) 92:548–51. doi: 10.1002/jmv.25722 PMC7228250 32096567

[102] Zhou P Yang XL Wang XG Hu B Zhang L Zhang W. A pneumonia outbreak associated with a new coronavirus of probable bat origin. Nature (2020) 579:270–3. doi: 10.1038/s41586-020-2012-7 PMC7095418 32015507

[103] Ottenhoff TH Ab BK Van Embden JD Thole JE Kiessling R. The recombinant 65-kD heat shock protein of mycobacterium bovis bacillus calmette-Guerin/M. tuberculosis is a target molecule for CD4+ cytotoxic T lymphocytes that lyse human monocytes. J Exp Med (1988) 168:1947–52. doi: 10.1084/jem.168.5.1947 PMC2189100 2903217

[104] Finotti P. Sequence similarity of HSP65 of mycobacterium bovis BCG with SARS-CoV-2 spike and nuclear proteins: may it predict an antigen-dependent immune protection of BCG against COVID-19? Cell Stress Chaperones (2021) 27:37–43. doi: 10.1007/s12192-021-01244-y PMC8577642 34755305

[105] Eggenhuizen PJ Ng BH Chang J Fell AL Cheong RMY Wong WY. BCG Vaccine derived peptides induce SARS-CoV-2 T cell cross-reactivity. Front Immunol (2021) 12:692729. doi: 10.3389/fimmu.2021.692729 34421902 PMC8374943

[106] Tomita Y Sato R Ikeda T Sakagami T. BCG Vaccine may generate cross-reactive T cells against SARS-CoV-2: In silico analyses and a hypothesis. Vaccine (2020) 38:6352–6. doi: 10.1016/j.vaccine.2020.08.045 PMC7440160 32863070

[107] Cai C Peng Y Shen E Huang Q Chen Y Liu P. A comprehensive analysis of the efficacy and safety of COVID-19 vaccines. Mol Ther (2021) 29:2794–805. doi: 10.1016/j.ymthe.2021.08.001 PMC8342868 34365034

[108] Baden LR El Sahly HM Essink B Kotloff K Frey S Novak R. Efficacy and safety of the mRNA-1273 SARS-CoV-2 vaccine. N Engl J Med (2021) 384:403–16. doi: 10.1056/NEJMoa2035389 PMC7787219 33378609

[109] Voysey M Clemens SAC Madhi SA Weckx LY Folegatti PM Aley PK. Safety and efficacy of the ChAdOx1 nCoV-19 vaccine (AZD1222) against SARS-CoV-2: an interim analysis of four randomised controlled trials in Brazil, south Africa, and the UK. Lancet (London England) (2021) 397:99–111. doi: 10.1016/S0140-6736(20)32661-1 33306989 PMC7723445

[110] Keech C Albert G Cho I Robertson A Reed P Neal S. Phase 1-2 trial of a SARS-CoV-2 recombinant spike protein nanoparticle vaccine. New Engl J Med (2020) 383:2320–32. doi: 10.1056/NEJMoa2026920 PMC7494251 32877576

[111] Xia S Zhang Y Wang Y Wang H Yang Y Gao GF. Safety and immunogenicity of an inactivated SARS-CoV-2 vaccine, BBIBP-CorV: a randomised, double-blind, placebo-controlled, phase 1/2 trial. Lancet Infect Dis (2021) 21:39–51. doi: 10.1016/S1473-3099(20)30831-8 33069281 PMC7561304

[112] Zhu FC Li YH Guan XH Hou LH Wang WJ Li JX. Safety, tolerability, and immunogenicity of a recombinant adenovirus type-5 vectored COVID-19 vaccine: a dose-escalation, open-label, non-randomised, first-in-human trial. Lancet (2020) 395:1845–54. doi: 10.1016/S0140-6736(20)31208-3 PMC7255193 32450106

[113] Sadoff J Gray G Vandebosch A Cardenas V Shukarev G Grinsztejn B. Safety and efficacy of single-dose Ad26.COV2.S vaccine against covid-19. New Engl J Med (2021) 384:2187–201. doi: 10.1056/NEJMoa2101544 PMC8220996 33882225

[114] Hviid A Hansen JV Thiesson EM Wohlfahrt J. Association of AZD1222 and BNT162b2 COVID-19 vaccination with thromboembolic and thrombocytopenic events in frontline personnel : A retrospective cohort study. Ann Internal Med (2022) 175:541–6. doi: 10.7326/M21-2452 PMC8805866 35103482

[115] Pottegård A Lund LC Karlstad Ø Dahl J Andersen M Hallas J. Arterial events, venous thromboembolism, thrombocytopenia, and bleeding after vaccination with Oxford-AstraZeneca ChAdOx1-s in Denmark and Norway: population based cohort study. Bmj (2021) 373:n1114. doi: 10.1136/bmj.n1114 33952445 PMC8097496

[116] Zhu F Jin P Zhu T Wang W Ye H Pan H. Safety and immunogenicity of a recombinant adenovirus type-5-vectored COVID-19 vaccine with a homologous prime-boost regimen in healthy participants aged 6 years and above: a randomised, double-blind, placebo-controlled, phase 2b trial. Clin Infect Dis (2022) 75:e783–e791. doi: 10.1093/cid/ciab845 34551104 PMC8522421

[117] Lazarus JV Ratzan SC Palayew A Gostin LO Larson HJ Rabin K. A global survey of potential acceptance of a COVID-19 vaccine. Nat Med (2021) 27:225–8. doi: 10.1038/s41591-020-1124-9 PMC7573523 33082575

[118] Figa Z Temesgen T Zemeskel AG Ganta M Alemu A Abebe M. Acceptance of COVID-19 vaccine among healthcare workers in Africa, systematic review and meta-analysis. Public Health Pract (Oxford England) (2022) 4:100343. doi: 10.1016/j.puhip.2022.100343 PMC9681992 36438628

[119] Dror AA Eisenbach N Taiber S Morozov NG Mizrachi M Zigron A. Vaccine hesitancy: the next challenge in the fight against COVID-19. Eur J Epidemiol (2020) 35:775–9. doi: 10.1007/s10654-020-00671-y PMC8851308 32785815

[120] Troiano G Nardi A. Vaccine hesitancy in the era of COVID-19. Public Health (2021) 194:245–51. doi: 10.1016/j.puhe.2021.02.025 PMC7931735 33965796

[121] Larson H Karafilakis E Antoniadou E Baka A Kramarz P. Vaccine hesitancy among healthcare workers and their patients in Europe. Vaccine (2015) 34:5013–20. doi: 10.1016/j.vaccine.2016.08.029 27576074

[122] Miller A Reandelar MJ Fasciglione K Roumenova V Otazu GH. Correlation between universal BCG vaccination policy and reduced morbidity and mortality for COVID-19: an epidemiological study. medRxiv (2020), 20042937. doi: 10.1101/2020.03.24.20042937

[123] Curtis N Sparrow A Ghebreyesus TA Netea MG. Considering BCG vaccination to reduce the impact of COVID-19. Lancet (London England) (2020) 395:1545–6. doi: 10.1016/S0140-6736(20)31025-4 PMC7252177 32359402

[124] Gupta PK. New disease old vaccine: Is recombinant BCG vaccine an answer for COVID-19? Cell Immunol (2020) 356:104187–. doi: 10.1016/j.cellimm.2020.104187 PMC7386780 32745670

[125] Ebina-Shibuya R Horita N Namkoong H Kaneko T. Current national policies for infant universal bacille calmette-guérin vaccination were associated with lower mortality from coronavirus disease 2019. Clin Exp Vaccine Res (2020) 9:179–82. doi: 10.7774/cevr.2020.9.2.179 PMC7445316 32864376

[126] Czajka H Zapolnik P Krzych Ł Kmiecik W Stopyra L Nowakowska A. A multi-center, randomised, double-blind, placebo-controlled phase III clinical trial evaluating the impact of BCG re-vaccination on the incidence and severity of SARS-CoV-2 infections among symptomatic healthcare professionals during the COVID-19 pandemic in Poland-first results. Vaccines (Basel) (2022) 10:314. doi: 10.3390/vaccines10020314 35214772 PMC8879775

[127] Upton CM van Wijk RC Mockeliunas L Simonsson USH McHarry K van den Hoogen G. Safety and efficacy of BCG re-vaccination in relation to COVID-19 morbidity in healthcare workers: A double-blind, randomised, controlled, phase 3 trial. EClinicalMedicine (2022) 48:101414. doi: 10.1016/j.eclinm.2022.101414 35582122 PMC9098089

[128] Khan N Downey J Sanz J Kaufmann E Blankenhaus B Pacis A. M. tuberculosis reprograms hematopoietic stem cells to limit myelopoiesis and impair trained immunity. Cell (2020) 183:752–70.e22. doi: 10.1016/j.cell.2020.09.062 33125891 PMC7599081

